# Isolation of Shiga toxin-producing *Escherichia coli* from raw milk in Kermanshah, Iran

**Published:** 2013-09

**Authors:** Pantea Mohammadi, Ramin Abiri, Mansour Rezaei, Siavosh Salmanzadeh-Ahrabi

**Affiliations:** 1 Medical biology Research Center, University of Medical Sciences, Kermanshah, Iran; 2 Department of Microbiology, Faculty of Medicine, University of Medical Sciences, Kermanshah, Iran; 3 Department of Statistics and Epidemiology, Faculty of Health, University of Medical Sciences, Kermanshah, Iran; 4 Department of Microbiology, Alzahra University, Tehran, Iran

**Keywords:** STEC, Antimicrobial resistance, raw milk, Iran

## Abstract

**Background and Objectives:**

Infectious diarrhoeal diseases are great problem throughout the world and are responsible for considerable morbidity and mortality. Shiga toxin-producing *Escherichia coli* (STEC) is a major cause of gastroenteritis that may be complicated by hemorrhagic colitis (HC) or the hemolytic uremic syndrome (HUS), which is the main cause of acute renal failure in children. Food-borne outbreaks associated with Shiga toxin-producing *Escherichia coli* have been well documented worldwide.

The aim of this study was to investigate the prevalence of Shiga toxin-producing *Escherichia coli* (STEC) strains in raw milk samples.

**Materials and Methods:**

Raw milk samples collected from various cow farms in Kermanshah, Iran during June - September 2009 were investigated for STEC using PCR targeting *stx1* and *stx2* and then *eaeA*.

**Results:**

Of 206 samples, 36 (17.47%) were contaminated with STEC. STEC isolates harbored 56.41% and 43.59% *stx*_*2*_ and *stx*_*1*_ gene respectively. In antibiotic resistance test, all strains were sensitive to ceftazidime, cefepime, gentamicin, imipenem and ciprofloxacin. 23.08% of isolates were resistat to tetracycline, and 38.5% of them showed intermediate sensitvity to cephalothin.

**Conclusions:**

The high presence of STEC in raw milk confirms the important role of raw milk as putative vehicle of infection to human. Moreover, this study suggests that the development of antibiotic resistant STEC must be a major concern in Iran and more studies are needed to identify the prevalence of STEC in other food samples.

## INTRODUCTION

Shiga toxin-producing *Escherichia coli* (STEC), also called Verotoxin-producing *E. coli* (VTEC), are a subgroup of *Escherichia coli* capable of producing one or two potent toxins called Shiga toxin (Stx_1_, Stx_2_) or Verotoxin (VT_1_, VT_2_) and may also possess additional putative virulence factors such as intimin which is responsible for intimate attachment of STEC to the intestinal epithelial cells, causing attaching and effacing (A/E) lesions in the intestinal mucosa (1, 2). This pathotype is a major cause of gastroenteritis that may be complicated by hemorrhagic colitis (HC) or the hemolytic uremic syndrome (HUS), which is the main cause of acute renal failure in children (2–4). Food-borne outbreaks associated with STEC have been well documented worldwide. STEC O157:H7 was reported as the causative agent of a series of outbreaks occurring primarily in Canada, Japan, the US and the UK (3, 5, 6). Cattle are considered the primary reservoir of both O157 and non-O157 STEC strains (7). Transmission of this food-borne pathogens occur through consumption of under cooked meat, unpasteurized dairy products, vegetables or water contaminated by ruminant feces. Contact with infected animal or human has also been documented (8, 9).

One of the most contentious areas in the management of STEC infections lies in the possible effect of antimicrobials on the natural history of the infections. Because antimicrobials may lyse bacterial cell walls, thereby liberating Shiga toxins (10, 11), and/or cause increased expression of Shiga toxin genes in vivo (12), they are not recommend for treating STEC O157:H7 infections. However recent studies suggest that some antimicrobials, if administered early in the course of infection, may prevent disease progression to HUS (13, 14). Although STEC infections are not aggressively treated with antimicrobial therapy and many isolates are susceptible to numerous antimicrobials, recent reports indicate that antimicrobial resistance of STEC is on the rise (15, 16).

Enhanced nutritional quantities, task and health benefits have all been advocated as reasons for increased interest in raw milk consumption. Although some comprehensive studies have been conducted in developed countries about raw milk contamination with STEC, unfortunately we still lack relevant data from Asia, and especially from the Middle-East. Thus, the objective of this study was to determine the prevalence and virulence profile of STEC isolated from raw milk in Iran, as well as to examine antimicrobial resistance profiles of isolates.

## MATERIALS AND METHODS

### Samples

From 22nd June to 22nd September 2009, a total of 206 bulk-tank milk samples were collected from 135 cow farms with a total of approximately 6,000 animals in Kermanshah. These farms ranged in size from 10 to 500 animals. The samples were placed on ice and transported immediately to the laboratory.

### Bacterial culture

25 ml of the milk sample (about 500 ml) was cultured in 225 ml of modified EC broth containing cefexime (0.05 mgL^-1^, Daana Pharmaceutical Co.) and then incubated overnight at 37°C. A portion of EC broth was spread on a plate of MacConkey agar which was incubated overnight at 37°C.

### DNA extraction

A loopfull of bacteria from the primary streak were collected and DNA was extracted according to the previously described protocol (17). The supernatant was used in PCR reactions targeting *stx*_*1*_ and *stx*_*2*_ as described below.

### PCR primer and reaction conditions

**Fig. 1:**
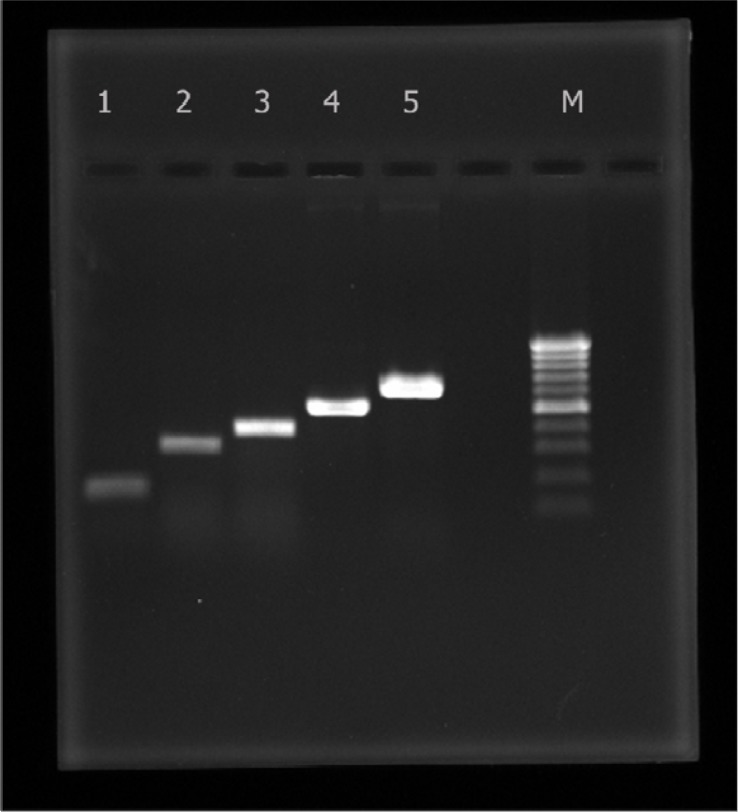
Agarose gel electrophoresis of *stx*_*1*_, *stx*_*2*_, *eaeA*, *rfbO157* and *fliCh7* PCR products from lane 1 to lane 5 respectively. Lane M: 100 bp molecular size marker.

Amplification of bacterial DNA was performed in thermal cycler (Bio Rad) using 25 µl volumes containing 5 µl of the prepared sample supernatant; lx reaction buffer; 0.5 µM of each primer; 0.2 mM of each dNTP; 1.5 mM MgCl_2_ and 1.2 U of Taq DNA polymerase (Cinnagen Co.). After amplification, 10 µl of each sample was analyzed by 1.5% agarose gel electrophoresis for the detection of positive samples. A number of colonies ranging from 30-90 were tested in order to find the pure colony or colonies responsible for the positive results in the first PCR, and then DNA extracts from responsible colonies that were confirmed as *E.coli* with biochemical tests were examined for the following genes: *eaeA*, *rfbO157* and *fliCh7* (18). Primers and cycling condition are listed in Table 1. For all amplification reactions, the mixture was heated at 96°C for 4 min prior to thermocycling. The mixture was held at 72°C for 6 min after the final cycle before cooling at 4°C. The following *E.coli* strains were included as conrol in each PCR run: STEC, ATCC 43890 (*stx1*) and ATCC 43889 (*stx2*); and EPEC; ATCC 43887 (*eaeA* and *bfpA*). Agarose gel electrophoresis of *stx*_*1*_, *stx*_*2*_, *eaeA*, *rfbO157* and *fliCh7* PCR products is seen in Fig. 1, (19–22).

**Table 1:** Primers and cycling condition.

Target	Oligonuceotide sequence (5′-3)	PCR condition	No. of cycles	Fragment size(bp)	Reference
*Stx1*	GAAGAGTCCGTGGGATTACG AGCGATGCAGCTATTAATAA	94°C, 20s; 50°C, 20s; 70°C, 12s	32	130	Pollard et al. (19)
*Stx2*	ACCGTTTTTCAGATTTTGACACATA TACACAGGAGCAGTTTCAGACAGT	94°C, 20s; 61.3°C, 20s; 70°C, 12s	32	298	Svenungsson et al. (20)
*rfbo157*	AAGATTGCGCTGAAGCCTTTG CATTGGCATCGTGTGGACAG	94°C, 20s; 64.8°C, 20s; 70°C, 12s	32	479	Desmarchelier et al. (21)
*fliCh7*	GCGCTGTCGAGTTCTATCGAGC CAACGGTGACTTTATCGCCATTCC	94°C, 20s; 69.8°C, 20s; 70°C, 12s	32	625	Gannon et al. (22)
*eaeA*	CACACGAATAAACTGACTAAAATG AAAAACGCTGACCCGCACCTAAAT	94°C, 20s; 61.3°C, 20s; 70°C, 12s	32	376	Svenungsson et al. (20)

### Antimicrobial testing

The STEC strains were tested for antibiotic resistance using the disk diffusion method (23). Antibiotic disks (Mast) used were ceftazidime (30 µg), cefepime (30 µg), erythromycin (15 µg), gentamicin (10 µg), cephalothin (30 µg), imipenem (10 µg), ciprofloxacin (5 µg) and tetracycline (30 µg).

## RESULTS

### Prevalence of STEC in raw milk sample

Among the 206 milk samples, 36 (17.47%) were positive for STEC, and 39 strains were isolated.

### Virulence genes

PCR showed that 22 (56.41%) strains carried *stx*_*2*_ gene and 17 (43.59%) strains possessed *stx*_*1*_ gene. All the strains were *eae*-negative, and STEC O157:H7 was not seen.

### Antimicrobial resistance among STEC strains

While all the strains were sensitive to ceftazidime, cefepime, gentamicin, imipenem and ciprofloxacin, thirty (76.92%) of 39 STEC were sensitive to tetracycline and the rest (23.8%) were resistant to it. In addition, fifteen (38.5%) of the strains.had intermediate sensitivity to cephalothin. Although multidrug resistance was not seen, 60% of strains that had intermediate sensivity to cephalothin were resistant to tetracycline.

## DISCUSSION

For the rapid and sensitive detection of STEC from clinical and food samples, PCR has proven to be of great diagnostic value (24, 25). Cultivation of food and stool material in liquid medium or on plates may increase the number of bacteria and may therefore assist in the detection of STEC which are present in low numbers or in a physiologically stressed state. For this reason, PCR test was carried out after the enrichment of milk samples in EC broth and cultivation of a portion of the EC broth on MacConkey agar.

We showed that the STEC prevalence in raw milk samples was 17.47%. The samples were collected during the summer months, which had been associated with a peak in the number of cows which are carring STEC (26). Therefore, milk contamination with STEC may be much less frequent at other times of the year.

Parisi et al. (27) reported a lower STEC prevalence (5.7%) in raw milk in Apuila region (SE Italy). Similarly, the STEC prevalences of raw milk in Ontario and Germany were reported to be 0.87% and 3.9%, respectively (28, 29). In the previous report from Fance, 21% of the 205 samples of raw milk were positive for STEC, indicating a prevalence level similar to our data (30). Numerous factors are likely to contribute to the variation observed such as geographical location, season, farm size, number of animals on the farm, hygiene, farm management practices, variation in sampling, variation in types of samples evaluated, and differences in detection methodologies used.

In our study, the majority of the STEC isolates carried *stx*_*2*_ gene; however, the presence of this gene proved to be variable in different regions (24, 27, 30–32). All of our STEC strains were *eae-*negative, which may be caused the low pathogencity of isolates. However, it should be noted that production of intimin is not essential for pathogenesis because a number of sporadic cases of HUS were caused by *eae*-negative non-O157 STEC strains. For example, STEC strains lacking *eae* gene were responsible for human illness outbreaks in the United States and Australia (33–35). This study did not reveal any instance of O157:H7 STEC, which is in accord with some data from Canada, Australia and the UK (Scotland) (21, 29, 36). However, there are several reports of presence of O157:H7 STEC in raw milk in other studies (37, 38). There was no report about prevalence of O157 and non-O157 STEC in raw milk in Iran, but the greatest majority of research carried out on fecal samples of human only reported isolation of non-O157 STEC. For example Aslani and his colleague found that 0.7% of 3268 faecal samples from randomly selected inhabitants of two provinces in the northern region were positive for STEC, however none of the isolates belonged to O157:H7 serotype. Conversely, Salmanzadeh-ahrabi et al. (39) investigated STEC in Tehranian children with acute diarrhea and matched controls without diarrhoea reported isolation of STEC from 15% of patients and 2% of controls, which 7 out of 30 STEC strains isolated from patients were O157:H7 (40–42).

In addition to therapeutic use of antimicrobials in human, the use of antimicrobial agents for disease prevention and growth promotion of animals has been a common practice on farms. It leads to a selection of antimicrobial resistance among commonsals in intestinal tracts of livestock animals, which poses potential negative clinical implications (43). Thus, continued surveillance of emerging antimicrobial resistance among zoonotic food-borne pathogens, including STEC, is required to ensure public health. Based on the research done on STEC recovered from poultry, cattle, swine and humans in Pennsylvania, 83 (30%) of the isolates were resistant to tetracycline (44). In other studies in India and Iran, resistance to tetracycline was 23.8% and 10%, respectively (45, 46). Resistance to this antibiotic also was observed in our results. 87.18% of our isolates showed resistance to erythromycin, while all strains isolated from meat in Iran were resistant to it (46). In addition, 38.5% of intermediate sensitivity to cephalothin should be paid attention in treatment schedule.

In summary, our data revealed that non-O157:H7 STEC are more prevalent, and the high presence of STEC in raw milk confirms the important role of raw milk as putative vehicle of infection to human. Moreover, this study suggests that the development of antibiotic resistant STEC must be a major concern in Iran and more studies are needed to identify the prevalence of STEC in other food samples.
